# A Rare Presentation of Serratia marcescens Endocarditis

**DOI:** 10.7759/cureus.54670

**Published:** 2024-02-22

**Authors:** Fahad Alshughaithry, Mohammed Bahatheg, Abdulrahman Barri, Musaed Albawardi

**Affiliations:** 1 Internal Medicine, King Saud University College of Medicine, Riyadh, SAU; 2 Internal Medicine, King Saud University Medical City, Riyadh, SAU

**Keywords:** spice organism, serratia, serratia marcescens, serratia marcescens endocarditis, infective endocarditis

## Abstract

*Serratia marcescens* is uncommon and rarely causes bacterial endocarditis. It can follow a rapid and progressive course with high mortality. Here, we present the case of a 27-year-old gentleman with bacterial endocarditis secondary to *S. marcescens* who was successfully treated medically.

## Introduction

*Serratia marcescens* is an opportunistic gram-negative bacillus associated with nosocomial infections, intravenous drug abuse, immunosuppression, and indwelling catheters and is known for causing urinary tract infections, respiratory tract infections, and soft tissue infections [1,2]. It is rarely associated with a primary invasive infection but presents as an opportunistic infection in immunocompromised hosts [2]. Infective endocarditis due to *S. marcescens* is extremely rare and can follow a rapid and devastating course [3]. The most implicated risk factor for endocarditis with *Serratia* is intravenous drug use [4]. An analysis of 63 cases of endocarditis secondary to *Serratia* was found in Embase, and PubMed revealed a predilection of aortic and mitral valve involvement despite the association of intravenous drug use in 60% of cases [5].

## Case presentation

A previously healthy 27-year-old gentleman presented to the hospital with a history of fever reaching 39°C for nine days. Initially, the fever was intermittent, usually at night, and responded to paracetamol. It then became persistent throughout the day despite paracetamol associated with night sweats and shivering, with a 10 kg weight loss in nine days from decreased oral intake. He went to a private medical center where he was diagnosed with a viral-like illness and was given cefuroxime, which he used for three days with no improvement. He then presented to our hospital. Initial workup revealed gram-negative bacteremia from two sets within four hours. He was started empirically on 1 g of meropenem intravenously every eight hours with subsequent resolution of fever.

**Figure 1:**
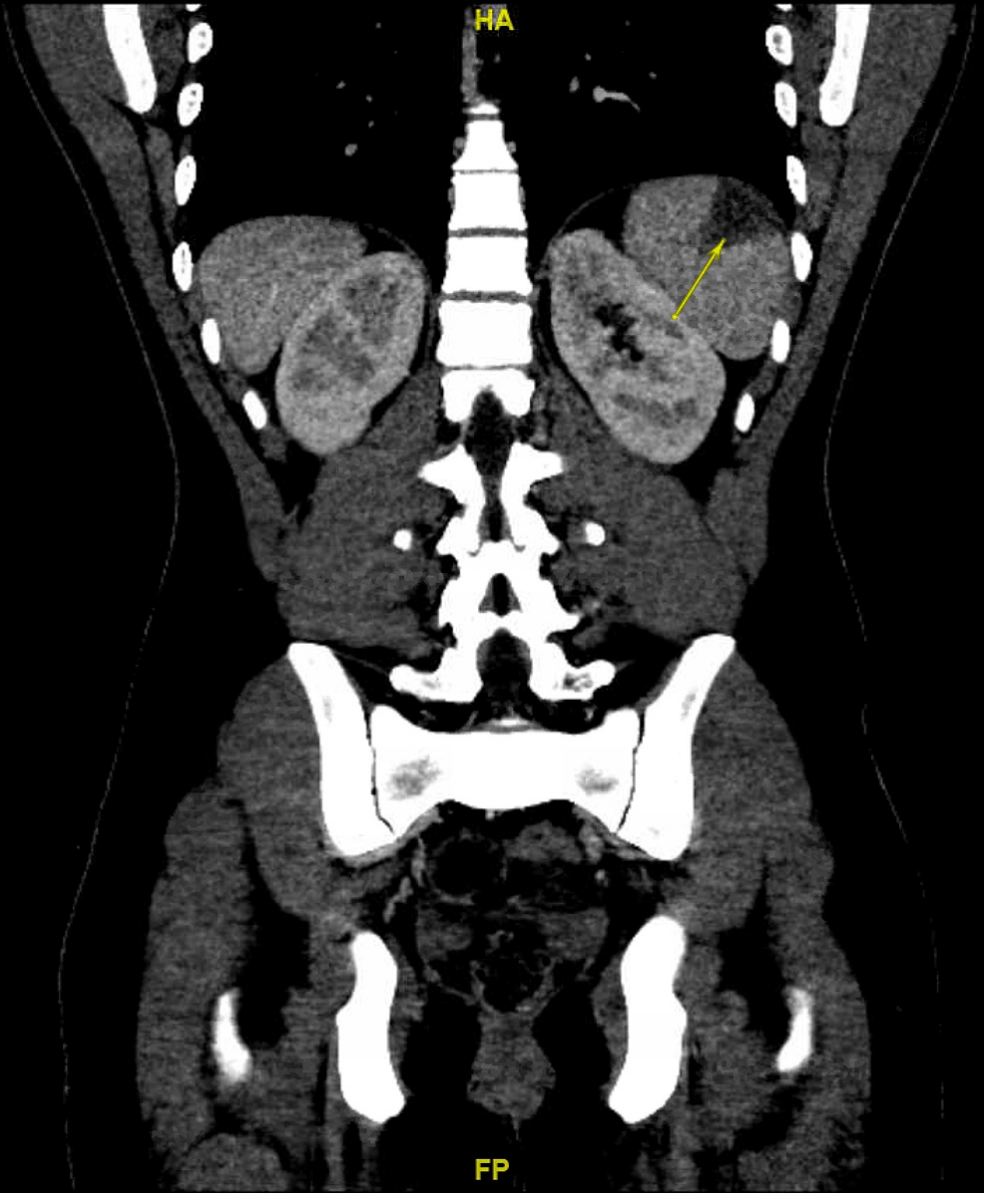
CT scan of the abdomen showing a wedge-shaped infarction in the spleen (yellow arrow).

Given that the patient had no apparent source of infection, a CT of the abdomen was done and showed embolic lesions in the right kidney and spleen causing wedge-shaped infarction (Figure 1). The final result of the blood culture isolated *S. marcescens* resistant to amoxicillin/clavulanate, both first and third-generation cephalosporin, and sensitive to fluoroquinolones, trimethoprim-sulfamethoxazole, cefepime, and carbapenems.

**Figure 2:**
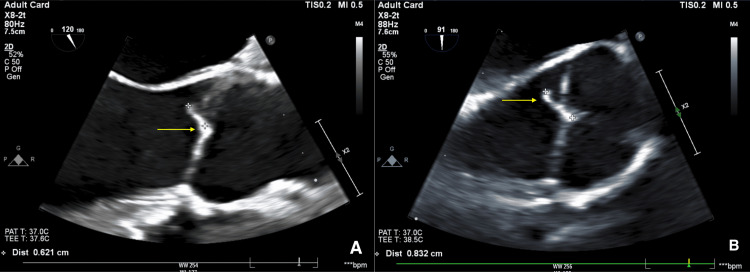
Transesophageal echocardiography showing moderate-sized mobile vegetation on the non‐coronary cusp of the aortic valve seen in two views (yellow arrows), measuring 8 mm in length (plane B).

The finding of renal cortical and splenic infarctions and *S. marcescens* bacteremia raised suspicion of bacterial endocarditis. Hence, transesophageal echocardiography was performed and revealed moderate-sized mobile vegetation on the non‐coronary cusp of the aortic valve, measuring 8 mm in length. Additionally, mild aortic regurgitation and no aortic root abscess or vegetation in other valves were identified (Figure 2). He denied any risk factors for infective endocarditis such as intravenous lines or intravenous drug or substance abuse, recent dental procedures, or recent surgical procedures. human immunodeficiency virus testing was performed and revealed a negative result on two separate occasions.

Definitive therapy with cefepime 1 g every eight hours intravenously for four weeks was given and meropenem was discontinued. A repeated transesophageal echocardiogram showed an interval resolution of the previously demonstrated aortic valve vegetation, and the patient was discharged home in stable condition.

## Discussion

*S. marcescens* is an opportunistic gram-negative bacillus well-known for its propensity to cause nosocomial infections, particularly in immunocompromised individuals or those with indwelling catheters [6]. Although its association with urinary tract infections, respiratory tract infections, and soft tissue infections is well-documented, primary invasive infections are infrequent.

Infective endocarditis caused by *S. marcescens* is uncommon but can have a rapid and devastating course if left untreated [3]. While intravenous drug use is a commonly identified risk factor for this condition, our patient did not exhibit any traditional risk factors. This serves as a reminder of the importance of considering less common pathogens in patients without typical predisposing factors.

This case highlights some important points-the patient presented with non-specific symptoms that could be attributed to systemic infection. Despite an apparent lack of traditional risk factors, he was ultimately found to have *S. marcescens* bacteremia and infective endocarditis. This emphasizes the importance of prompt, accurate diagnosis and initiating appropriate antibiotic therapy, even in patients without apparent predispositions.

Given the rarity of *S. marcescens* infective endocarditis, the medical literature provides limited guidance on optimal treatment approaches. However, the successful and favorable outcome observed in this case supports empirically administering a targeted antibiotic regimen based on susceptibility testing of the isolated pathogen. Multiple case reports have reported similar susceptibility profiles and used similar empiric antimicrobial therapy [1,7]. Nonetheless, further studies are warranted to establish evidence-based treatment guidelines and optimize the management of *S. marcescens* infective endocarditis.

## Conclusions

*S. marcescens* endocarditis is an uncommon and potentially life-threatening condition. This case report underscores the significance of considering atypical pathogens in patients presenting with infective endocarditis, even without traditional risk factors. Timely diagnosis, appropriate antimicrobial therapy, and close monitoring are crucial for favorable outcomes in such cases.
